# Ruthenium Complexes as NO Donors for Vascular Relaxation Induction

**DOI:** 10.3390/molecules19079628

**Published:** 2014-07-07

**Authors:** Renata Galvão de Lima, Bruno Rodrigues Silva, Roberto Santana da Silva, Lusiane Maria Bendhack

**Affiliations:** 1 Faculdade de Ciências Integradas do Pontal, Universidade Federal de Uberlândia, Ituiutaba 38304-402, MG, Brazil; E-Mail: renatagalvao@pontal.ufu.br; 2 Departamento de Física e Química, Faculdade de Ciências Farmacêuticas de Ribeirão Preto, Universidade de São Paulo, Ribeirão Preto 14040-903, SP, Brazil; E-Mails: brunofarmaco@gmail.com (B.R.S.); silva@usp.br (R.S.S.)

**Keywords:** nitric oxide, ruthenium-derived complexes, vasodilation, blood pressure control, soluble guanylyl cyclase, potassium channels

## Abstract

Nitric oxide (NO) donors are substances that can release NO. Vascular relaxation induction is among the several functions of NO, and the administration of NO donors is a pharmacological alternative to treat hypertension. This review will focus on the physicochemical description of ruthenium-derived NO donor complexes that release NO via reduction and light stimulation. In particular, we will discuss the complexes synthesized by our research group over the last ten years, and we will focus on the vasodilation and arterial pressure control elicited by these complexes. Soluble guanylyl cyclase (sGC) and potassium channels are the main targets of the NO species released from the inorganic compounds. We will consider the importance of the chemical structure of the ruthenium complexes and their vascular effects.

## 1. Introduction

Nitric oxide (NO) has become one of the most important intercellular signaling molecules identified in the 1980s. Knowledge about its involvement in all kinds of physiological systems continues to expand. NO plays a role in platelet aggregation inhibition [1]; it also participates in the control of respiration [2], neurotransmission [3,4], hormone release [5], cell death [6], immune response [7], vascular tone [8,9,10], and blood pressure [11,12].

The formation, role, and mechanism of action of NO have been exhaustively reviewed [13,14,15]. The conversion of l-arginine to l-citrulline by the enzyme NO synthase (NOS) produces physiological NO. However, it is in the vascular smooth muscle that NO interacts with the ferrous state of the heme prosthetic center of the β_1_ subunit of the enzyme soluble guanylyl cyclase (sGC)—it activates sGC and consequently produces cyclic GMP (cGMP). The latter activates the cGMP-dependent kinase protein (GK), diminishing the cytosolic calcium concentration ([Ca^2+^]_c_) in the vascular smooth muscle cells (VSMC). This mechanism reduces the vascular resistance and helps to maintain the blood pressure at normal levels. 

NO is a relatively unstable molecule. It is produced at low concentrations and in the blood it is rapidly converted into nitrate and nitrite within 10 s of its formation [16]. This short half-life seems to be even lower in several cardiovascular diseases such as hypertension, because oxidative stress reduces NO availability. Several experimental hypertension models can simulate the impaired NO production/availability along with other vascular alterations that are typical of endothelial dysfunction [17]. Therefore, NO supplementation seems to be an interesting strategy to compensate for NO loss and could help ensure normal vasodilation and blood pressure control. In this context, some researchers have proposed that NO donor molecules constitute an effective tool to study this messenger. Authors have also suggested evaluating the different pharmacological characteristics of these molecules, to find out how they can impact the therapy of cardiovascular diseases.

Glyceryltrinitrate (GTN), obtained by Ascanio Sobrero in 1847, was one of the first NO donor molecules to be described. GTN is an organic molecule, a nitrate ester of glycerol. Despite its potent vasodilating and hypotensive effects, it causes side effects like headache and tolerance, which has limited its clinical application [18]. The classic nitrovasodilators organic nitrate and nitrite esters, including GTN, amyl nitrite, isosorbide dinitrate, and nicorandil, have long been used to treat cardiovascular diseases [19,20]. Sodium nitroprusside (SNP), an inorganic molecule, is yet another important and extensively studied NO donor. SNP is a metal nitrosyl complex composed of iron, cyanide groups, and a nitro moiety that together form a square bipyramidal complex capable of inducing potent hypotensive effect. Clinicians have employed SNP for decades, even though it releases highly cytotoxic cyanide [21]. 

At present, the development of exogenous NO donor agents such as ruthenium-derived metal nitrosyl complexes represents a strategy to reduce any possible side effects and cytotoxicity. Indeed, the ruthenium metal displays chemical characteristics that are similar to those of the iron metal present in the enzymes of biological systems [22]. In addition, nitrosyl ruthenium species seem to be good candidates for the treatment of cardiovascular diseases, because they are generally thermodynamically stable and NO-labile upon external stimulation [23]. 

Hence, this review will highlight the cellular mechanisms involved in the vasodilation prompted by NO and their relationship with the chemical structure of some ruthenium-derived complexes synthesized by our group. It will also discuss the chemical characteristics and vascular mechanisms of the classical NO donor SNP as well as other NO donors.

## 2. Nitric Oxide and Its Main Vasodilating Mechanism

### 2.1. NO Release and Vascular Biological Target

The amount and the site of NO release play an important part in the relaxation induced by NO donors. It is well known that NO is little bioavailable in the cellular milieu. The amount and the lifetime of the NO species released from the NO donors determine the pharmacological effects of this molecule. To induce vascular relaxation, the NO released from NO donors or produced by NO-synthases has to bind to the heme-protein sGC located in the cytosol of the vascular smooth muscle cells or activate potassium (K^+^) channels directly. 

### 2.2. Cellular Mechanisms for Vasodilation

The NO produced by the NO-synthase enzyme isoforms or the NO released by NO donors binds to sGC in the cytosol of the VSMC, to modulate the activity of sGC positively. The latter enzyme converts GTP to cGMP, which activates protein kinase G (GKIα). Swayze and Braun [24] have shown that GKIα alone is sufficient to activate K^+^ channels (BKCa) *in situ* following activation of the NO-cGMP signaling pathway. Activation of cGMP-dependent protein kinases induces vascular relaxation because it lowers the cytosolic Ca^2+^ concentration and promotes Ca^2+^ desensitization of the actin-myosin contractile system in the VSMC [25]. Furthermore, NO and K^+^ channels interact directly, which induces hyperpolarization, reduces cytosolic Ca^2+^ concentration, and improves vasodilation.

## 3. Sodium Nitroprusside and Its Effects on Vascular Tone and Arterial Pressure Control

Sodium nitroprusside (SNP, Na_2_[Fe(CN)_5_(NO)]·2H_2_O) was chemically characterized in the middle of the 19th century [26]. Its ability to lower the blood pressure has been known since 1929 [27]. SNP has been clinically employed as a hypotensive agent for over five decades [28], although only after the study reported by Furchgott *et al.* [8] its pharmacological effect has been attributed to ability of NO release. Regardless of pH and the photolysis wavelength (over a wide range), the aqua-complex [Fe(CN)_5_H_2_O]^2^^−^ and NO are the primary photochemical products from SNP in aqueous solution [29], as shown in Equation (1):

[Fe^II^(CN)_5_NO]^2^^−^ + H_2_O → [Fe^III^(CN)_5_H_2_O]^2^^−^ + NO (1)

On the other hand, it is currently known that NO formation from SNP is also accompanied by cyanide (CN^−^) release [30]. This leads to multiple effects including sGC activation and cell toxicity [31,32], and it constitutes an important limitation to the pharmacological use of SNP. In spite of CN^−^ release, SNP has long been a gold standard for the chemical development of new NO donors based on ruthenium complexes. Indeed, SNP is commonly employed as a reference drug when the pharmacological parameters (potency, maximum effect, time-course) of novel drugs are being investigated in terms of vasodilation and blood pressure control. In mammals, it is well known that the vasodilating/hypotensive response in mammals occurs readily after SNP injection [29,33,34]. Fast NO dissociation from [Fe(CN)_4_(NO)]^2^^−^ has been traced. [Fe(CN)_5_(NO)]^3^^−^ originates after CN^‑^ release in physiological pH [29,30] as shown in Equation (2):

[Fe^II^(CN)_5_NO]^3^^−^ ↔ [Fe(CN)_4_NO]^2^^−^ + CN^−^ (2)

Bates and co-workers have reported that in the presence of vascular tissue, SNP can release NO via a photochemical process or by decomposition [31]. Biological studies carried out in our research laboratories have confirmed the vasodilating/hypotensive effects of SNP. Our results indicated that SNP releases NO immediately, as measured by amperometric assay in solution. The NO level peaks in the beginning, with subsequent rapid decrease in its concentration within the following 200 s [35]. Addition of a specific fluorescence probe for NO, DAF-2DA, to the aortic slices exposed to SNP, reveals significantly increased fluorescence intensity (∆FI: 49.4% ± 4.0%; *n* = 5) [36]. The system takes 195 s to reach maximum rat aorta relaxation [35,36,37]. It is noteworthy that in the case of vascular tissues like the basilar artery, SNP releases NO into the intracellular medium [38,39].

Depending on the NO donor, the released NO species can trigger different action mechanisms, which will result in vasodilation anyway. NO species have been classified as radicalar NO (NO^0^) or nitroxyl (NO^−^ or HNO) species. Some years ago, NO^0^ was described as biological NO having sGC as the main target, whereas NO^−^ could originate from NO donors such as Angeli’s Salt, to activate K^+^ channels. Some current studies have reported that both types of NO can emerge in biological systems, but each of them activates types of K^+^ channels [40,41]. It is possible to distinguish between the different types of NO release by using pharmacological tools like selective scavengers, namely hydroxycobalamin for NO^0^ and l-cysteine for NO^−^/HNO [36,42].

Our vascular functional studies have indicated that SNP induces maximum vasodilating effect (101.2% ± 2.8%, *n* = 5) with high pharmacological potency in rat aorta (pD_2_: 8.25 ± 0.12, *n* = 5) [37]. Moreover, it releases both types of NO, NO^0^ and NO^−^ [36], in accordance with chemical studies reported by Olabe [30]. SNP contains the formally described nitrosonium (NO^+^) ligand, which may be redox-interconverted to the corresponding one-electron (NO) and two-electron (NO^−^/HNO) reduced bound species. 

SNP produces its vasodilating effect via sGC activation. This increases cGMP production and certainly activates the GK protein. In turn, this signaling activates K^+^ channels and the sarco/endoplasmic reticulum calcium-ATPase (SERCA) in the VSMC, which diminishes [Ca^2+^]_c_ and induces vascular relaxation [37,43,44]. However, SNP may also relax blood vessels through “cGMP-dependent” and “cGMP-independent” mechanisms [45]. Compared with normotensive rat aorta, SNP elicits a similar vasodilation mechanism in hypertensive rat aorta. Nevertheless, in the latter case the vasodilating potency of SNP is lower, because the SERCA activation induced by SNP is impaired [44]. The vasodilating effect induced by SNP is also impaired in vena cava from hypertensive rats, because the functional activity of the K^+^ channels is impaired, as well. Curiously, SERCA activation is not involved in the venorelaxation induced by SNP [46].

**Figure 1:**
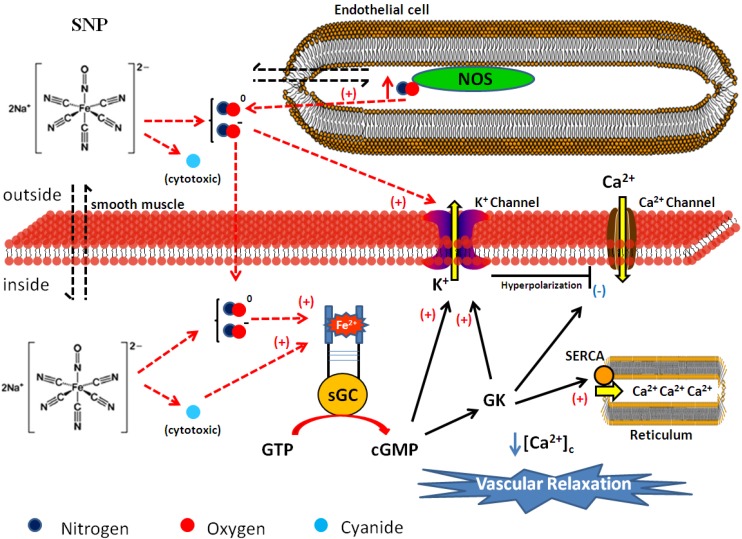
Proposed nitric oxide release and vasodilating mechanism prompted by sodium nitroprusside (SNP). The colored circles represent atoms in the chemical structure. Legend: NOS = Nitric Oxide Synthase, sGC = soluble guanylyl cyclase, GK = G Kinase Protein, SERCA = sarco/endoplasmic reticulum calcium-ATPase, Ca^2+^ = calcium, K^+^ = potassium, [Ca^2+^]_c_ = cytosolic calcium concentration.

Interestingly, SNP has been considered an endothelium-independent relaxant agent for many years; its effect has always been attributed to its direct action on the VSMC. Recently, our research group has demonstrated that SNP raises [Ca^2+^]_c_ in endothelial cells via activation of Ca^2+^ channels. This endothelial effect increases the NOS activity, thereby inducing NO production and potentiating the relaxant effect induced by SNP [47]. On the basis of all these findings, the mechanism proposed for the SNP-induced is the one shown in Figure 1.

Our *in vivo* analyses have attested that SNP induces a faster and greater hypotensive effect as compared with the ruthenium-based NO donor complex Terpy ([Ru(tpy)(NH.NHq)NO]^3+^) [34]. Compared with normotensive Wistar rats, SNP induces a stronger hypotensive effect in spontaneous hypertensive rats (SHR) and in normotensive rats. The effect is immediate, and the hypotensive effect is associated with increased heart rate in SHR and normotensive rats [48].

## 4. Development of Ruthenium-Derived NO Donor Complexes

Ruthenium compounds containing nitrogen oxide as one of their ligands generally exist as hexacoordinated species like [RuL_5_(NO_x_)]^n+^. Studies on such ruthenium complexes involve the reactivity of the coordinated nitrogen oxide ligands mainly, which include redox properties, photochemical reactivity, and kinetic aspects. The influence of co-ligands “**L**” on the [RuL_5_(NO)]^n+^ species is remarkable and will drive all the properties that are directly related to the nitrogen oxide ligands.

The compounds described in this work have been developed by our group and will be divided into two classes: (i) [Ru(N_4_)L(NO)]^n+^, which contains a tetraazacoordinated ligand as co-ligand (N_4_ is tetraazamacrocyclic ligand or phthalocyanine; n could be 2+, 1+, or 0); and (ii) [Ru(L)_x_L_y_NO]^n+^ (bpy refers to 2,2'-bipyridine or tpy refers to 2,2':6',2''-terpyridine). Both classes of compounds are stable in aqueous solution for at least 24 h. The present article will describe the physicochemical and the pharmacological properties of a specific class of ruthenium compounds.

### 4.1. Tetraazacyclotetradecane Ruthenium Complexes

**Figure 2:**
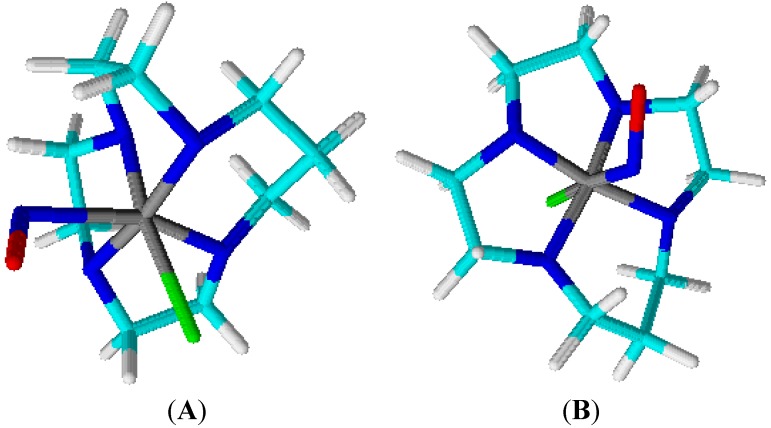
Nitrosyl macrocyclic ruthenium complexes structure. (**A**) *trans*-[RuCl([15]ane_4_)NO]^2+^ and (**B**) *trans*-[RuCl(cyclam)NO]^2+^. The colored circles represent atoms in the chemical structure (cyan: carbon; blue: nitrogen; green: chlorine; red: oxygen; white: hydrogen and gray: ruthenium).

The tetraazacyclotetradecane ruthenium complexes synthesized by our group bear 1,4,8,11-tetrazacyclotetradecane (cyclam) or 1,4,8,12-tetrazacyclopentadecane ([15]aneN_4_ ) ligands coordinated to ruthenium(II) or (III) compounds (Figure 2). Both species are quite soluble in aqueous solution; the macrocycle (MAC) chelates the metal ion, which in principle offers extra stability to the complex as compared with the open chain ligand [49]. Generally, the ruthenium compound bearing these ligands are isolated as [RuCl_2_(MAC)]Cl. When MAC refers to [15] aneN_4_, only the species with *trans-*geometry occurs. As for MAC corresponding to cyclam, the *cis/trans-*geometries are achieved. We have successfully prepared both [RuCl_2_(MAC)]Cl complexes in the *trans*-geometry, because the resulting ruthenium(III) complex is inert to the chloro ligand. The latter phenomenon is partly due to the affinity of the ruthenium(III) ion for charged negative ligands and partly due to hydrogen bonding between MAC and the bound chloride in *trans-*[RuCl_2_(MAC)]Cl [50,51]. Bearing in mind that the chelate ligand MAC on the ruthenium complex is inert, and that the chloro ligand becomes labile after reduction of the metal ion to ruthenium(II), it is possible to synthesize and isolate *trans-*[Ru(NO)Cl(MAC)]X (X = Cl^−^ or PF_6_^−^) in good yields and purity.

**Scheme 1:**
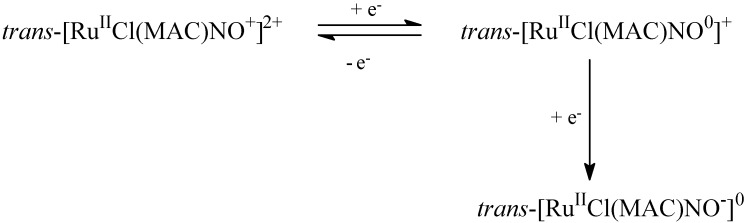
Electrochemical process involved in nitrosyl macrocyclic ruthenium complex.

Coordinated NO can assume three different forms depending on the oxidation state, namely nitroxyl for NO^−^, nitric oxide for NO^0^, and nitrosyl for NO^+^. For *trans-*[Ru(NO)Cl(MAC)]^+^, the nitrogen oxide ligand has nitrosyl character, as verified by FTIR spectral analysis. Regarding the structure of [Ru-NO]^3+^, the ν_(NO)_ values range from 1,700 to 2,000 cm^−1^ [52,53,54,55]. The ν(NO) stretching for *trans*-[RuCl(cyclam)NO]^2+^ and *trans*-[RuCl([15]ane_4_)NO]^2+^ appears at 1,864 and 1,860 cm^−1^, respectively. Both values indicate that a high degree of positive charge resides on the coordinated nitrosyl, so it is better to describe ruthenium as ruthenium(II). The electrochemical characterization of *trans*-[RuCl(cyclam)NO]^2+^ and *trans*-[RuCl([15]ane_4_)NO]^2+^ shows that both macrocyclic ruthenium complexes could undergo reduction in the aqueous solution window potential via two electrochemical steps, as illustrated in Scheme 1. 

**Scheme 2:**
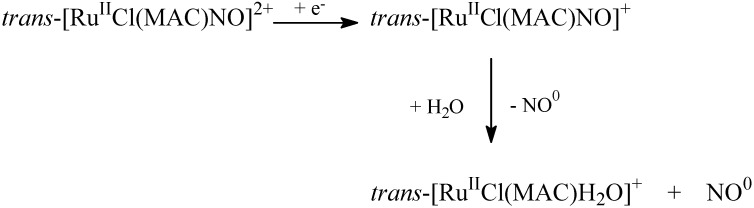
Kinetic process related to the electron reduction of nitrosyl macrocyclic ruthenium complex.

The highest reduction process is almost reversible in the cyclic voltammetry timescale and refers to NO^+/0^. The second reduction process is irreversible and corresponds to the NO^0/^^−^ process [52,53,54,55]. Using 0.1 M KCl as electrolyte, the mono-reduction electrochemical process for the *trans*-[RuCl(cyclam)NO]^2+^ and *trans*-[RuCl([15]ane_4_)NO]^2+^complexes takes place at 122 and 280 mV *vs.* Ag/AgCl, respectively. At very low cyclic voltammetry scan rate, the NO^+/0^ couple is almost irreversible, which suggests that a chemical reaction follows the electrochemical step. The controlled electrolyses reduction process conducted in the presence of the NO sensor provides the best explanation for the process: NO release occurs after the reduction process (Scheme 2). Therefore, these kinds of compounds are applicable as nitric oxide delivery agents for pharmacological assays. Indeed, some reducing biomolecules like norepinephrine or ascorbic acid can promote such reduction process [35].

Bonaventura *et al.* [35] have reported pharmacological studies involving reduction of *trans*-[RuCl([15]ane_4_)NO]^2+^ using norepinephrine, a well-known vasoconstrictor, as the biological reducing agent. These studies will be properly discussed later in this paper. From a chemical viewpoint, the reduction of a nitrosyl ruthenium complex depends on the formation of a supramolecule between norepinephrine and the ruthenium complex, mediated by a phosphate bridge. The phosphate ion interacts with *trans*-[RuCl([15]ane_4_)NO]^2+^ by hydrogen bonding and plays a crucial role in the reduction of the nitrosyl ruthenium complex [23].

**Scheme 3:**
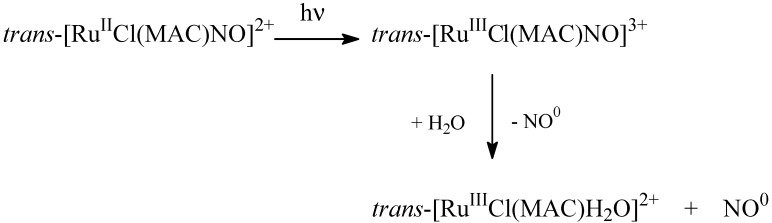
General photochemical pathway related to nitrosyl macrocyclic ruthenium complex.

In addition to NO release via chemical reduction, it is also possible to control NO release by light irradiation. Based on the UV-visible spectral analysis and measurement with the NO sensor, we have described the photochemical process for *trans-*[Ru(NO)Cl(MAC)]^+^ as illustrated in Scheme 3.

The photoproduct of the *trans*-[RuCl(cyclam)NO]^2+^ and *trans*-[RuCl([15]ane_4_)NO]^2+^complexes obtained by light irradiation at 355 nm is aqua(macrocyclic)ruthenium(III), with consequent NO release. The measured NO quantum yield (ϕNO) is 0.1 and 0.6 eisntein mol^−1^ for the *trans*-[RuCl(cyclam)NO]^2+^ and *trans*-[RuCl([15]ane_4_)NO]^2+^complexes, respectively. The contribution of the nitrosyl ligand to the lowest unoccupied molecular orbital (LUMO) best explains the different quantum yields of these complexes. Indeed, LUMO seems to be larger in *trans*-[RuCl([15]ane_4_)NO]^2+^ than in *trans*-[RuCl(cyclam)NO]^2+^ [56]. Observation of the chemical and the photochemical properties as well as the attainment of different pharmacological results for the various macrocyclic nitrosyl ruthenium complexes are a consequence of the macrocyclic ring size [56].

In the rat aorta tissue, *trans*-[RuCl([15]ane_4_)NO]^2+^ releases NO by chemical reduction. The release of NO from this reduced compound is slower than the release of NO from SNP. However, this ruthenium compound sustains high levels of NO release, which reaches a maximum at 5 min [35]. As shown previously, in artery pre-contracted with norepinephrine, the complex *trans*-[RuCl([15]ane_4_)NO]^2+^ induces relaxation in a concentration-dependent way. The time necessary to achieve maximal relaxation is 595 s, which is longer as compared with the time that SNP takes to induce relaxation [35]. However, Bonaventura [35] demonstrated that the relaxing effect of *trans*-[RuCl([15]ane_4_)NO]^2+^ is completely blocked in aorta pre-contracted with high KCl concentration (60 mM). At this large concentration, KCl induces membrane depolarization, voltage-operated Ca^2+^ channels activation, and Ca^2+^ influx, to stimulate the contractile machinery.

To study vascular relaxation, it is necessary to contract the vessels. The choice of the contractile agent is very important, since it will remain in contact with the vessel during the course of the relaxation experiment. Norepinephrine stimulates contraction through α-adrenoceptor activation and intracellular as well extracellular calcium mobilization, whereas KCl 60 mM induces contraction through extracellular calcium influx. It is important to consider that KCl at this extracellular concentration blocks the K^+^ channels, which play a pivotal role in the NO-induced vascular relaxation. We have also studied the relaxant effect of *trans*-[RuCl([15]ane_4_)NO]^2+^ in the prostaglandin F_2α_ (PGF_2α_)-induced contraction [57]. In denuded-endothelium aortic ring contracted with PGF_2α_, the photo-induction of the complex *trans*-[RuCl([15]ane_4_)NO]^2+^ with UV light induces complete relaxation in 50 s. These results suggest that this relaxation stems from NO release from preformed endogenous NO stores upon UV light irradiation. Increasing concentrations of KCl (as the pre-contractile agent) decreases the relaxation induced by nitrosothiols NO donors (GSNO and NACysNO) as compared with phenylephrine-contracted aorta [58].

The low cytotoxic characteristics of ruthenium-derived complexes are well known [59,60]. The *trans*-[RuCl([15]aneN_4_)NO]^2+^ complex is not toxic to the VSMC in the concentration that it induces maximum relaxation of denuded rat aorta.

#### 4.1.1. Vasodilating Effect in Normotensive and Hypertensive Rat Vessels

Several ruthenium-derived complexes induce relaxation in isolated vessels of normotensive and hypertensive rats. However, the relaxation induced by *trans*-[RuCl(15-aneN_4_)NO]^2+^ is less potent than the vascular relaxation induced by SNP. Interestingly, the efficacy is similar between these complexes. The *bis*-oxonol sensitive probe (DIBAC2) can detect changes in the membrane potential of rat aorta smooth muscle cells. This probe has revealed that cell membranes from renal hypertensive 2K-1C rat aorta are more depolarized (−55 mV) than 2K normotensive rat aorta cell membranes (−65 mV) [61]. In 2K-1C aorta cells, a KCl concentration of only 10 mM is necessary to induce complete depolarization as compared with the concentration of 40 mM required in the case of 2K aorta cells. An electrophysiological investigation into the cells membrane has shown that the resting potential is more electronegative in 2K than in 2K-1C aorta cells membrane, and that the hyperpolarization induced by acetylcholine is lower in 2K-1C than in 2K [62].

The ruthenium-derived complex *trans*-[Rucl([15]-aneN_4_)NO]^2+^ has led to intriguing findings. Selective (4-aminopyridine, apamin, iberiotoxin, glibenclamide) and also non-selective blockers of K^+^ channels (TEA) do not affect the relaxation induced by *trans*-[Rucl([15]-aneN_4_)NO]^2+^ in 2K-1C rat aorta. This could be the reason for the impaired relaxation observed in this hypertension model. On the other hand, all the studied K^+^ channel blockers abate the *trans*-[Rucl([15]-aneN_4_)NO]^2+^-induced relaxation of normotensive 2K rat aorta [63]. 

**Figure 3:**
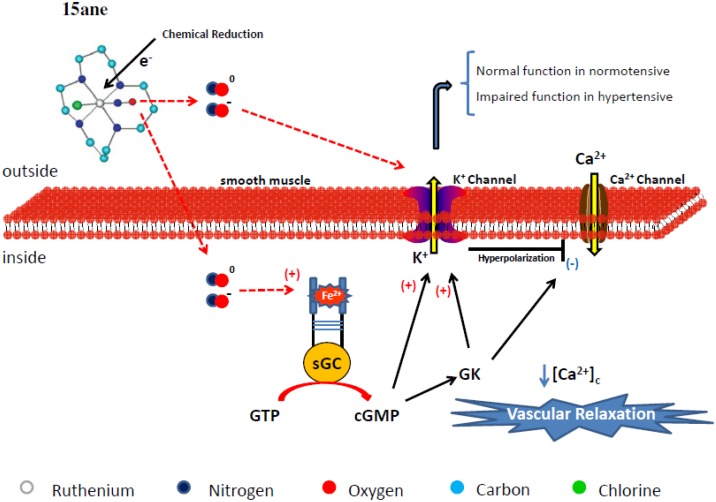
Proposed nitric oxide release and cellular mechanisms involved in the vasodilation promoted by *trans*-[RuCl(15-aneN_4_)NO]^+^ (15ane). The colored circles represent atoms in the chemical structure. The hydrogen atom has been omitted in the structure. Legend: sGC = soluble guanylyl cyclase, GK = G Kinase Protein, Ca^2+^ = calcium, K^+^ = potassium, [Ca^2+^]_c_ = cytosolic calcium concentration.

The vascular functional studies have indicated that *trans*-[Rucl([15]-aneN_4_)NO]^2+^ releases both types of NO, NO^0^ and NO^−^, and its vasodilating effect originates from K^+^ channel activation in the cGMP-dependent and -independent pathways, which diminish [Ca^2+^]_c_ and therefore induce vascular relaxation. However, K^+^ channel activation in the hypertension model seems to be impaired as compared with normotensive rat aorta. On the basis of these results, Figure 3 depicts the mechanism proposed for the vasodilatation induced by *trans*-[Rucl([15]-aneN_4_)NO]^2+^.

#### 4.1.2. Effects of the Macrocyclic Ruthenium Complexes on Arterial Pressure Control

Although no *in vitro* functional studies on the *trans*-[RuCl(cyclam)NO]^2+^ complex exists, Marcondes *et al.* [64] have reported how this complex affects the blood pressure *in vivo*. These authors found that, compared with SNP, *trans*-[RuCl(cyclam)NO]^2+^ induces 20 times longer hypotensive effect in both normotensive and hypertensive rats. They also found that sGC inhibitor or NO scavenger completely inhibits this effect.

On the other hand, our research group has observed that the hypotensive effect prompted by *trans*-[Rucl([15]-aneN_4_)NO]^2+^ depends on the severity of the hypertensive state. In severe renal hypertensive rats (2K-1C), the *trans*-[Rucl([15]-aneN_4_)NO]^2+^ induces a more pronounced effect than in mild hypertensive rats [65]. Comparison between two doses of *trans*-[Rucl([15]-aneN_4_)NO]^2+^, namely 0.1 mM/kg and 10 mM/kg, reveals that the lower dose does not have hypotensive effect on normotensive or moderate hypertensive rats, but it impacts severe hypertensive rats. The higher dose of the NO donor *trans*-[Rucl([15]-aneN_4_)NO]^2+^ reduces the mean arterial pressure of all hypertensive rats. In normotensive 2K rats, both doses of *trans*-[RuCl(15-aneN_4_)NO]^2+^ elicit a much lower hypotensive effect. To induce the hypotensive effect, *trans*-[RuCl(15-aneN_4_)NO]^2+^ requires higher doses as compared with SNP. 

Considering the series *trans*-[RuCl(mac)NO]^2+^ and *trans*-[Ru(NH_3_)_4_(L)NO]^3+^, it is possible to correlate the rate of NO release with the stability of the reduced [Ru(II)-NO°] compounds. This stability will depend on the electron density of the metal centers and on the nature of the ancillary ligands. In these series, the kinetics and thermodynamic properties of NO are sensitive to the ligand present in the *trans* position in the coordination sphere [64,66]. In this context, our group aimed to develop a new class of nitrosyl ruthenium complexes that would allow us to modulate the NO release elicited by co-ligands with π-acceptor character.

### 4.2. Polypyridine Ruthenium Complexes

**Figure 4:**
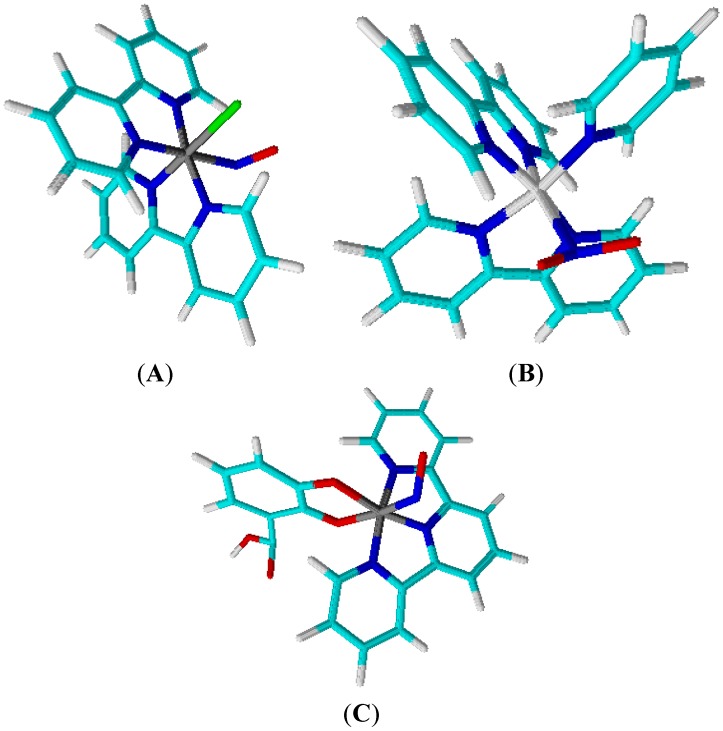
Chemical structure of polipyridines ruthenium complexes (**A**) *cis*-[RuCl(bpy)_2_NO]^2+^, (**B**) *cis*-[Ru(bpy)_2_(py)NO]^3+^ and (**C**) [Ru(tpy)(NH.NHq)NO]^3+^. The colored circles represent atoms in the chemical structure (cyan: Carbon; blue: Nitrogen; green: Chlorine; red: Oxygen; white: Hydrogen and gray: Ruthenium).

*Cis-*[Ru(bpy)_x_L_y_NO_z_]^n+^ (L = 2,2'-bipyridine, 2,2':6',2''-terpyridine, or pyridine-like ligands) will represent the polypyridine ruthenium complexes described in this paper. When these pyridine derivative ligands coordinate to ruthenium(II), they generally establish back-bonding with the metal ion. On the other hand, the nitrogen oxide ligand (NO_z_) is described as nitrosyl species (NO^+^) or Nitrite (NO_2_^−^) bound to ruthenium(II). This work will discuss two classes of these compounds, named *cis-*[Ru(bpy)_2_L(NO_z_)]^n+^ (*n* = 3+, 2+, 1+, 0) and [Ru(tpy)L’(NO_z_)]^n+^ (L' = 2,2'-bipyridine or 1,2-benzoquinonediamine) (Figure 4). The nature of NO_z_ depends on the pH of the solution; at pH > 4.0, the ruthenium species is normally ascribed as nitro ligand coordinated to ruthenium(II).

**Figure 5:**
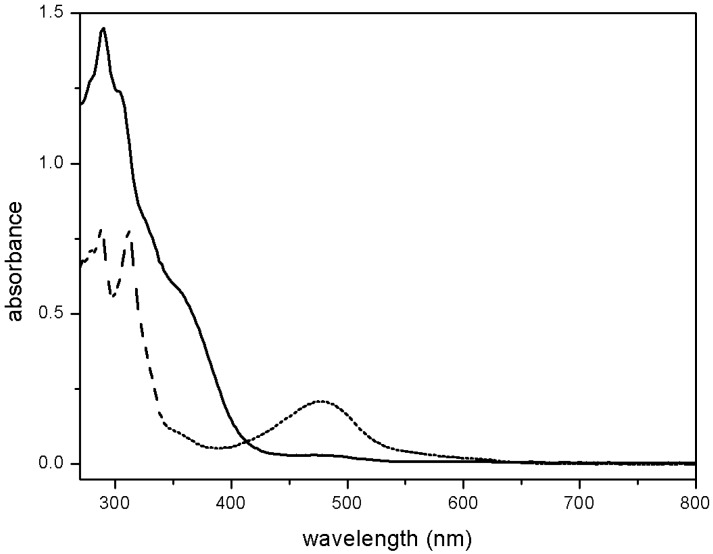
UV-visible electronic spectra of [RuNO_2_(tpy)(bpy)]^2+^ (dashed line) and [Ru(tpy)(bpy)NO]^3+^ (solid line) in aqueous solution.

The nitrosyl polipyridine ruthenium complex displays UV-visible spectrum with intense bands in the ultraviolet region [67,68,69,70]. These bands are due to intraligand transition originated from unsaturated ligands and to metal ligand charge transfer (MLCT) bands dπ(Ru^II^)-π*(polypyridine + NO^+^). No bands arise in the visible region [71]. In addition to the intraligand bands, the nitro species presents bands in the region of 450 nm, characterized as MLCT dπ(Ru^II^)-π*( polypyridine). Figure 5 brings the electronic spectrum of representative complexes of ruthenium polypyridine species.

Electrochemical studies conducted on these nitrosyl bipyridine species reveal two redox processes between +1 to −1 V *vs*. Ag/AgCl, as previously described for tetraazamacrocyclic compounds in this paper. Cyclic voltammetry results and FTIR observations evidence that the electron density under ruthenium(II) influences the NO^+/0^ process and depends on the back-bonding of co-ligands. Table 1 lists the NO^+/0^ reduction potential and the ν_(NO)_ stretching. Apparently, the σ- and π-donor character of “L” in *cis-*[Ru(bpy)_2_L(NO)]^2+^ lowers the reduction potential of the nitrosyl ligand, as observed for L = Cl^−^ as compared with the pyridine (py), 4-picoline (4-pic), and 4-acetylpyridine (4-acpy) ligands, which exert π-acceptor character only [52,53,72,73]. A similar correlation is true for the [Ru(tpy)L_n_NO]^n+^ complex [55].

**Table 1:** Infrared data and NO^+/0^ redox potential for nitrosyl polypyridine ruthenium complexes.

Compounds	ν(NO) cm^−1 a^	E_1/2_ NO^+/^NO^0^ (V) *vs.* Ferrocene ^b^
*cis*-[Ru^II^(bpy)_2_(NO)Cl]^2+^	1940	–0.21
*cis*-[Ru^II^(bpy)_2_(py)(NO)]^3+^	1944	0.12
*cis*-[Ru^II^(bpy)_2_(4-pic)(NO]^3+^	1947	0.14
*cis*-[Ru^II^(bpy)_2_(4-acpy)(NO)]^3+^	1943	0.16
[Ru^II^Cl_2_(tpy)(NO)]^3+^	1870	–0.51^c^
[Ru^II^(tpy)(bpy)(NO)]^3+^	1944	+0.053
[Ru^II^(tpy)(NH.NHq)(NO)]^3+^	1888	-

a: KBr pellets; b: Acetonitrile solvent; Hirano *et al.*, 2001 [72].

**Scheme 4:**
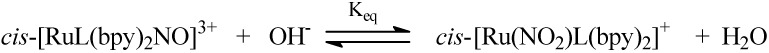
Interconversion of nitrosyl-nitrite coordinated to ruthenium(II).

The electron density under nitrosyl also influences its nucleophilic character [74]. This is easy to verify upon varying the pH. Scheme 4 describes the reactivity of representative species of the nitrosyl bipyridine ruthenium complex. The rate equilibrium constant (K_eq_) depends on the L that exists in *cis-*[Ru(bpy)LNO]^n+^.

The Keq values measured for the 4-pic, py, and 4-acpy complexes are 3.8 × 10^20^, 1.6 × 10^21^, and 3.2 × 10^21^ L^2^ mol^−^^2^, respectively. Keq increases with the π-acceptor capacity of the L ligand, which suggests that competition regarding back-bonding of the metal results in low electronic density on the nitrosyl group [53].

Depending on the irradiated light wavelength, the nitrosyl bipyridine ruthenium complexes are also photochemically active and release NO. To avoid nitrite formation as a result of the nucleophilic attack of OH^−^, photolysis of polypyridyl ruthenium complexes has to be carried out in buffer solution pH 2.0. Studies on the photoreactivity of the nitrosyl complex reveal a change in the UV-Visible spectrum; an NO-sensor aids quantification of the released NO. For all the polypyridyl nitrosyl ruthenium complexes irradiated with light of 355 nm, the absorption in the region of 330 nm (MLCT band attributed to dπ(Ru^II^) → π*(NO^+^) transition) decreases, while absorption in the visible region (400 to 500 nm) intensifies. Our group has suggested that the photochemical pathway followed by the polypyridyl nitrosyl ruthenium complexes under ultraviolet irradiation involves formation of the Ru-NO^0^ species in aqueous solution. Due to the low affinity between Ru(II) and NO^0^, NO release takes place, and Ru^II^-H_2_O arises as photoproduct [55,75,76].

As in the case of the NO^+/0^ redox potential data, our results indicated that the series of polypyridyl ruthenium complex affords higher ϕNO values during NO photorelease. This is consistent with the π-acceptor character of the ligand L (Table 2).

**Table 2:** Quantum yield (ϕNO) values for the nitrosyl polypyridine ruthenium complexes after flash photolysis at 355 nm and pH= 2.01 in trifluoroacetate buffer solution.

Compounds	ϕNO mol einstein ^−1^
*cis*-[Ru^II^(bpy)_2_(NO)Cl]^2+^	0.98 *
*cis*-[Ru^II^(bpy)_2_(py)(NO)]^3+^	0.16
*cis*-[Ru^II^(bpy)_2_(4-pic)(NO]^3+^	0.17
*cis*-[Ru^II^(bpy)_2_(4-acpy)(NO)]^3+^	0.07
[Ru^II^(tpy)(bpy)(NO)]^3+^	0.14
[Ru^II^(tpy)(NH.NHq)(NO)]^3+^	0.47

* pH = 5.7 phosphate buffer solution.

The low chemical stability in physiological pH limits the pharmacological use of bipyridine ruthenium species. Therefore, we have hypothesized that it is possible to employ nitro ruthenium species as a source of NO. Apparently, by means of a different photochemical mechanism, *cis-*[Ru(bpy)_x_L(NO_2_)]^n+^ is also photochemically reactive and produces NO under visible and ultraviolet light irradiation (Table 3) [77,78].

**Table 3:** Quantum yield (ϕNO) values of nitrosyl polypyridine ruthenium complexes after flash photolysis at 355 nm and pH = 7.4 in phosphate buffer solution.

Compounds	ϕNO mol eistein^−1^
*cis*-[Ru^II^(NO_2_)(bpy)_2_(py)]^+^	0.007
*cis*-[Ru^II^(NO_2_)(bpy)_2_(4-pic)]^+^	0.009
*cis*-[Ru^II^(NO_2_)(bpy)_2_(pz)]^+^	0.037
[Ru^II^(NO_2_)(tpy)(bpy)]^+^	0.036

The maximum quantum yield measured for *cis-*[Ru(bpy)_x_L(NO_2_)]^n+^ is around 4%. The suggested photochemical pathway involves homolytic cleavage of coordinated nitrite, to generate NO [77,78].

#### 4.2.1. RUNOCL

*cis*-[RuCl(bpy)_2_(NO)](PF_6_) (RUNOCL) is a metal complex consisting of ruthenium and bypiridine ligands, bearing chloride and nitrosyl groups in the axial positions of ruthenium. The main chemical characteristic of this complex is its irradiation-dependent NO release. This represents a singular advantage regarding controlled NO release, because only irradiation stimulates this complex. Apart from being very important in the field of photodynamic therapy, NO release from this structure contributes to a better understanding of how NO affects the vascular tone during experimental studies. After NO release, the reaction mediated by light produces the aqua complex, which does not seem to be toxic to the VSMC at the applied concentration [43].

Using the specific probe for [Ca^2+^]_c_, fluo-3 acetoxymethyl ester (Fluo-3 AM), in the VSMC isolated from rat aorta, it is possible to verify that the RUNOCL complex diminishes [Ca^2+^]_c_ (60.0% ± 10.0%; *n* = 4) in relation to the control (100%). The NO released from RUNOCL activates sGC sensitive to the selective inhibitor ODQ and K^+^ channels sensitive to TEA, reducing the [Ca^2+^]_c_ in the VSMC to 81.0% ± 5.0% (*n* = 4) and 79.0% ± 6.4% (*n* = 4), respectively. In addition, the combination of ODQ and TEA abolishes the decreased [Ca^2+^]_c_ in the VSMC more effectively (97.0% ± 3.5%; *n* = 4). Taken together, these results suggest that the NO released from RUNOCL can activate sGC and K^+^ channels, markedly lowering [Ca^2+^]_c_ [43].

Using the photo-induction technique and a visible light system with λ > 380 nm, evaluation of the vasodilating effect induced by RUNOCL in rat aortic ring attests that RUNOCL elicits relaxation under light. The maximum effect is 101.2% ± 3.7%, with pD_2_: 6.62 ± 0.16 (*n* = 7). This effect does not happen in the absence of light irradiation [79]. At the maximum concentration, 1630 s is the time-course that is necessary to reach the maximum relaxation induced by RUNOCL [80]. The sGC inhibition with ODQ reduces the maximum relaxation and pD_2_ to 30.1% ± 1.6% and to 6.35 ± 0.05, respectively (*n* = 4). Moreover, in the presence of light irradiation, RUNOCL increases the cGMP content in aortic tissue from 63.13 ± 0.45 fmol/μg of protein to 70.56 ± 4.64 fmol/μg of protein (*n* = 4). In the presence of K^+^ channel blockers, TEA diminishes the potency of RUNOCL to 5.32 ± 0.10 (*n* = 5), and iberiotoxin (large conductance Ca^2+^-dependent K^+^ channel blocker) reduces its maximum effect to 60.7% ± 3.4% (*n* = 5) [79]. 

**Figure 6:**
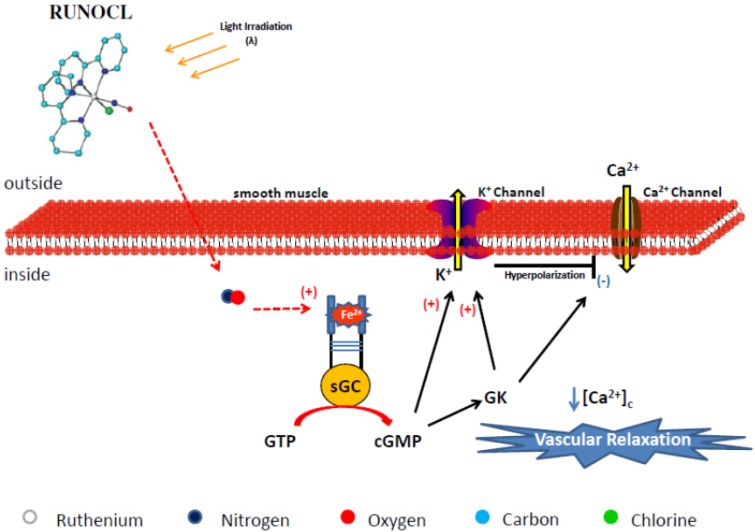
Proposed nitric oxide release and vasodilation mechanism elicited by *cis-*[RuCl(bpy)_2_(NO)](PF_6_) (RUNOCL). The colored circles represent atoms in the chemical structure. The hydrogen atom has been omitted in the structure. Legend: sGC = soluble guanylyl cyclase, GK = G Kinase Protein, Ca^2+^ = calcium, K^+^ = potassium, [Ca^2+^]_c_ = cytosolic calcium concentration.

In summary, these studies have indicated that the NO released from RUNOCL exerts its vasodilating effect by K^+^ channel activation via the cGMP-dependent pathway, which decreases [Ca^2+^]_c_ and induces vascular relaxation. On the basis of these results, Figure 6 illustrates the proposed mechanisms for the vasodilation induced by RUNOCL.

#### 4.2.2. RuBPY

*cis*-[RuNO_2_(bpy)_2_(py)](PF_6_) (RuBPY) is another metal complex that bears ruthenium, bypiridine ligands, and a nitrite group. The main chemical characteristic of this complex is its ability to release NO in the presence of the vascular tissue only. Basically, RuBPY differs from RUNOCL by the presence of nitrite (NO_2_) rather than NO in the molecular structure. Using a fluorescence probe specific for NO, DAF-2DA, it is possible to observe that the cytosolic NO concentration ([NO]_c_) in aortic slices increases significantly, as seen from the difference in the fluorescence intensity (∆FI) after exposure to RuBPY (∆FI: 1645.14 ± 388.30; *n* = 5). However, in the presence of ODQ, ∆FI decreases to 366.88 ± 120.3 (*n* = 7). Therefore, if we bear in mind that RuBPY only releases NO in the presence of rat aorta ring, it is possible to infer that the sGC-dependent mechanism converts NO_2_ from RuBPY to NO. The sGC inhibitor ODQ abolishes such NO_2_ conversion to NO [37]. 

As a pharmacological tool, ODQ oxidizes the sGC heme group, a potential center for NO_2_ reduction to NO. Researchers are extremely interested in NO_2_ conversion to NO [81]. NO originated from NO_2_ activates sGC, raising the cGMP concentration and inducing vasodilation. The pharmacological potency of RuBPY is 6.54 ± 0.07 (*n* = 5); the time-course is 240 s. However, sGC inhibition by ODQ reduces the vasodilation induced by RuBPY from 104.4% ± 1.1% to 38.0% ± 3.6% (*n* = 8) [37].

**Figure 7:**
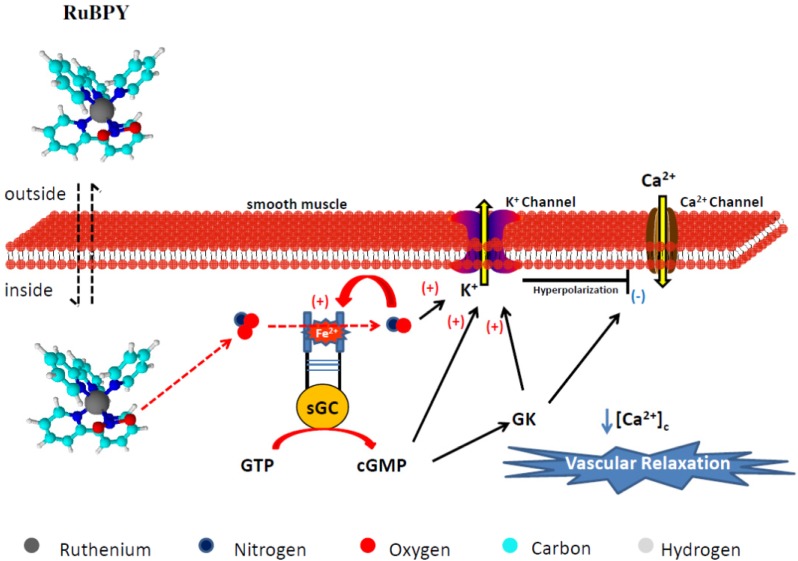
Proposed nitric oxide release and vasodilation mechanism elicited by *cis*-[RuCl(bpy)_2_(py)NO_2_](PF_6_) (RuBPY). The colored circles represent atoms in the chemical structure. Legend: sGC = soluble guanylyl cyclase, GK = G Kinase Protein, Ca^2+^ = calcium, K^+^ = potassium, [Ca^2+^]_c_ = cytosolic calcium concentration.

RuBPY releases radicalar NO species only. The NO^0^ released from RuBPY activates sGC, but it also seems to act directly on K^+^ channels, to induce VSMC membrane hyperpolarization in a sGC-independent way [36]. This process apparently differs from that verified in the case of RUNOCL. In accordance with these findings, Figure 7 details the proposed mechanisms for the vasodilation induced by RuBPY. 

#### 4.2.3. Terpy ([Ru(tpy)(NH.NHq)NO]^3+^)

The Terpy-induced vascular relaxation involves the release of both NO species, NO^0^ and NO^−^. It is important that NO released from the NO donors bind to sGC, to modulate the activity of this enzyme and produce cGMP. NO^0^ is the main type of NO binding to the sGC heme moiety, which in turn activates G-kinase. Among other proteins, G-kinase can activate K^+^ channels and/or SERCA.

The endogenous NO production by NO-synthase plays an important role in vascular tone control. sGC activation and cGMP production are believed to mediate this response. After that, NO is disposed in the form of inactive products such as nitrite and nitrate [82]. However, part of NO can modulate cell signaling by interacting with thiols and metals [83]. The distance from the point of synthesis or release and the cellular redox environment determine the biologically active concentration of NO [84]. Superoxide and subsequent peroxide production occur in many diseases such as hypertension [85]. In fact, hypertension is a multifactorial disease that involves many mechanisms including vascular endothelial dysfunction characterized by impaired NO bioavailability and increased reactive oxygen species production [17].

Impaired Terpy-induced relaxation occurs in renal hypertensive (2K-1C) rat aorta as compared with normotensive (2K) rat aorta. The cytosolic NO concentration is lower in 2K-1C than in 2K rat aorta, whereas superoxide concentration is larger in 2K-1C than in 2K rat aorta. The antioxidant vitamin C improves the vasodilating effect induced by Terpy in renal hypertensive rat aorta [86].

Considering that resistance vessels are relevant for arterial pressure control, investigating the vascular relaxation induced by Terpy in mesenteric resistance artery from hypertensive and normotensive rats is an interesting matter. In fact, this could explain the differences between the effects that Terpy exerts *in vivo* and *in vitro*. In contrast to the results obtained in conductance vessels, Araujo *et al.* (2013) [87] have shown similar Terpy-induced relaxation in mesenteric resistance artery of renal hypertensive and normotensive rats. Confocal microscopy images have shown that Terpy releases similar amounts of NO in normotensive rat aorta and aorta from SHR.

To study the vascular relaxation induced by several ruthenium-derived complexes while avoiding interference of endogenous NO in the response, the experiments are usually performed in endothelium-denuded artery. As reported by Bonaventura *et al.* [88], the complex Terpy can induce NO-synthase uncoupling and superoxide production.

#### 4.2.3.1. Vasodilating Effect in Normotensive and Hypertensive Rat Vessels

Upon activation by NO released from Terpy, sGC can produce a large amount of cGMP. Interestingly, the levels of cGMP produced by NO released from Terpy are similar in aorta isolated from normotensive (101.9% ± 1.5%, *n* = 5) and renal hypertensive rats (101.8% ± 2.0%, *n* = 5) (Bonaventura *et al.*, 2011). On the other hand, in normotensive sham-operated rats (2K), the relaxation is greater than that obtained in renal hypertensive (2K-1C) rat aorta. In the case of SHR aorta, Munhoz *et al.* have demonstrated that the relaxation induced by TERPY is not different from that observed in normotensive rat aorta (control) [48]. The vascular relaxation partially depends on sGC activation in SHR aorta [48].

In mesenteric resistance artery, 2K-1C and 2K do not differ in terms of the Terpy-induced relaxation [87]. The protein expression of GCs subunits (α_1_, β_1_) is similar in renal hypertensive and normotensive mesenteric arteries. G-kinase activated by cGMP can phosphorylate sarcoplasmic reticulum Ca^2+^-ATPase, which stimulates cytosolic Ca^2+^ uptake into this organelle and consequent relaxation. Callera and Bendhack [89] have shown that the SERCA function remains unaltered in 2K-1C rat aorta. In addition, Ca^2+^ uptake by SERCA does not seem to be involved in the Terpy-induced relaxation in 2K and 2K-1C rat aorta.

In the mesenteric artery, Terpy releases NO and activates cGMP-dependent protein reduction in K^+^ channels blockade and SERCA inhibition reduce in the same way during the relaxation induced by Terpy in 2K-1C and 2K mesenteric arteries [87]. However, SERCA inhibition in rat aorta does not inhibit the Terpy-induced relaxation [90].

**Figure 8:**
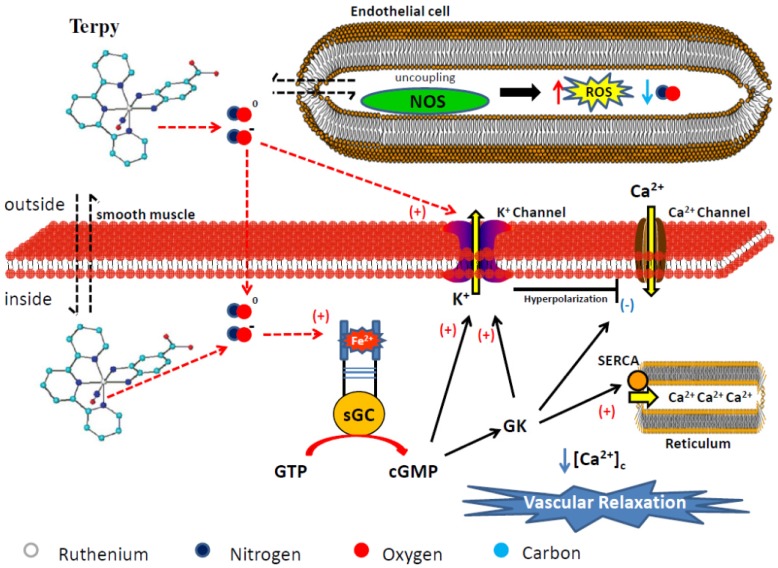
Proposed nitric oxide release and vasodilation mechanism induced by Terpy ([Ru(tpy)(NH.NHq)NO]^3+^). The colored circles represent atoms in the chemical structure. The hydrogen atom has been omitted in the structure. Legend: sGC = soluble guanylyl cyclase, GK = G Kinase Protein, SERCA = sarco/endoplasmic reticulum calcium-ATPase, Ca^2+^ = calcium, K^+^ = potassium, [Ca^2+^]_c_ = cytosolic calcium concentration.

NO produced by NO-synthase also acts as a vasodilator in cerebral arteries. Surprisingly, Terpy does not release NO in the cerebral rat basilar artery; it also fails to induce vascular relaxation in this artery [39]. In accordance with these findings, Figure 8 represents the partial mechanisms observed in each vessel for the vasodilation induced by Terpy.

#### 4.2.3.2. Effects of Terpy on Arterial Pressure Control

The mean arterial pressure depends directly on the vascular resistance and cardiac output. Despite the impaired vascular relaxation in 2K-1C rat aorta, the hypotensive effect is greater in 2K-1C than in 2K rats. The complexes 15ane and Terpy induce reduction in the mean arterial pressure in a dose-dependent way in both 2K-1C and 2K rats ([65] and [34], respectively). Terpy also prompts a hypotensive effect in SHR, as shown by Munhoz *et al.* [48]. The hypotensive effect induced by Terpy is slow; it does not lead to reflex tachycardia in neither SHR nor normotensive rats. The Terpy-induced hypotensive effect is more potent in SHR than in normotensive Wistar rats. As compared with SNP, Terpy induces hypotension less potently [48].

Hypertension is a multifactorial disease that involves several organ systems. Increased reactive oxygen species have been described at its onset and during its progression [91]. Endothelial dysfunction plays a significant role in hypertension, due to the increased production of endothelium-derived contractile substances such as reactive oxygen species.

### 4.3. NO Production by Visible Light Irradiation of Nitrosyl Ruthenium Complex

**Figure 9:**
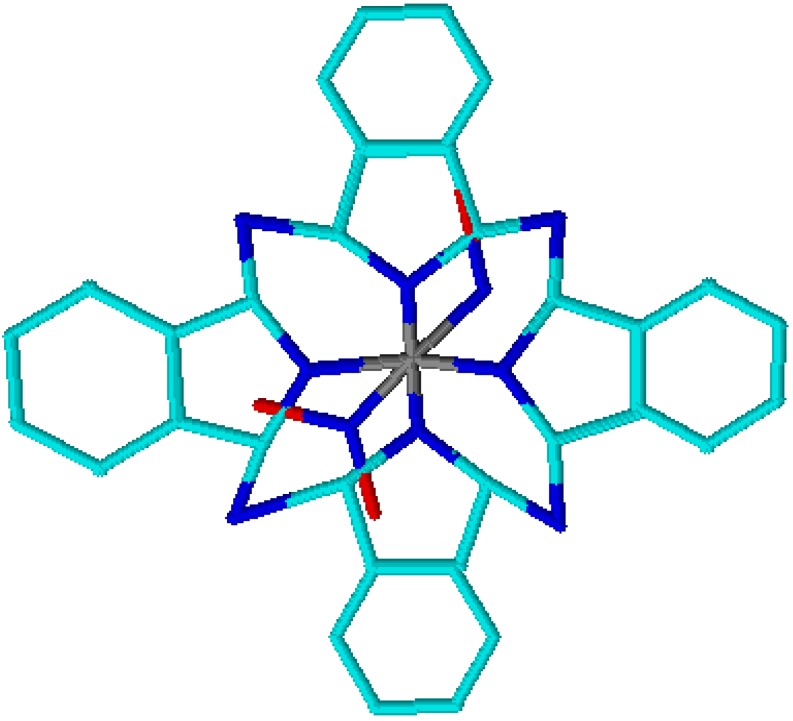
Chemical structure of the nitrosyl phthalocyanine ruthenium complex [Ru(NO)(NO_2_)pc]. The colored circles represent atoms in the chemical structure (cyan: carbon; blue: nitrogen; red: oxygen and gray: ruthenium).

The possibility of using nitrosyl ruthenium complexes as NO delivery agents via photolysis has motivated us to design species that exhibit strong light absorption within the therapeutic window. To the best of our knowledge, the synthesized binuclear [Ru_A_(L)(NH_3_)_4_(pz)Ru_B_(bpy)_2_NO]^5+^ (L = amin, py, 4-pic and 4-acpy) constitutes the first example of a ruthenium complex with such property. The fragment [Ru_A_(L)(NH_3_)_4_(pz)] works as an antenna: it absorbs light and photo-induces electron transfer to the second ruthenium fragment complex[Ru_B_(bpy)_2_NO]. The final process culminates in NO production [75,76]. The excited state lifetime observed upon irradiation with wavelength of 532 nm is in the order of picoseconds, which should account for the low quantum yield observed during photolysis of [Ru_A_(L)(NH_3_)_4_(pz)Ru_B_(bpy)_2_NO]^5+^. Binuclear complexes are a great concept on which to base the development of a coordination compound to serve as source of NO by photoinduced electron transfer. The synthesis of the compound [Ru(phthalocyanine)NO_2_NO] (designated [Ru(NO)(NO_2_)pc] [92]. hereafter, (Figure 9) is based on the same concept, and its photochemical and pharmacological properties have been described. Phthalocyanines have also proven to be efficient dyes for light absorption in the phototherapeutic window. Indeed, they constitute one of the second-generation photosensitizers used in photodynamic therapy (PDT) [92,93,94,95].

**Scheme 5:**
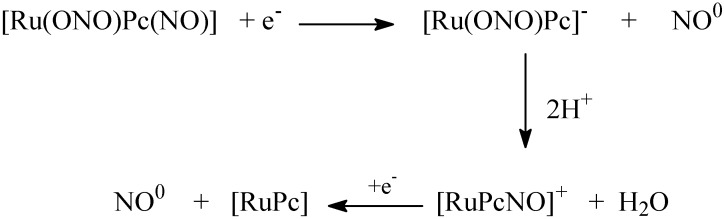
Kinetic process related to the electron reduction of nitrosyl phthalocyanine ruthenium complex.

The [Ru(NO)(NO_2_)pc] complex releases two NO molecules via a reduction process [78] or under irradiation with light at 670 nm. Scheme 5 describes the overall mechanism. Photolysis revealed that [Ru(NO)(NO_2_)pc] can also produce singlet oxygen, providing the opportunity to explore cytotoxicity aspects involving the synergism between NO and singlet oxygen [95]. Apparently, the biological mechanism involves reduction of the ruthenium species in the membrane, followed by NO release. We have proven this hypothesis from the vasodilation viewpoint. The [Ru(NO)(NO_2_)pc] complex induces vascular relaxation in the same way as other ruthenium-based NO donor agents [95].

## 5. General Aspects and Conclusions

The time-course necessary to reach the maximum effect, the maximum effect *per se*, the pharmacological potency, and the action mechanisms of NO donors have been evaluated. These pharmacological parameters indicate that the molecular structure of the compounds to which NO is coordinated provides specific characteristics such as the reduction potential (chemical or tissue-dependent) and excitation upon light irradiation, which can modulate the time (kinetic profile), the type, and the site of NO release. On the basis of our experience, employing ruthenium complexes with different molecular structures can contribute to achieving different pharmacological parameters as well as distinct final outcomes, although the NO is always the effector molecule underlying vasodilation. Over the years, these ruthenium-derived NO donor complexes have contributed to studying important vascular alterations in vessels of hypertensive rats such as K^+^ channel functions [44,63], increased reactive oxygen species production [86], and loss of caveolae [96] that can impair the vasodilation induced by NO.

The description of these complexes and their characteristics as NO donors can contribute to a better understanding of the activation of the vascular nitrergic pathway in healthy and diseased tissues. It can also facilitate the selection of specific molecules as prototypes for the development of drugs with potential therapeutic application.
